# Attentional focus modulates physiological response to arousal in a competitive putting task

**DOI:** 10.3389/fpsyg.2024.1497139

**Published:** 2024-11-06

**Authors:** Bobby Rawls, Victor Finomore

**Affiliations:** Rockefeller Neuroscience Institute, West Virginia University, Morgantown, WV, United States

**Keywords:** motor performance, competitive anxiety, external focus, heart rate variability, audiovisual distraction

## Abstract

Attentional focus during the execution of perceptual motor tasks has been shown to affect performance outcomes. The purpose of this study is to assess the physiological changes prompted by attentional focus in various levels of stress. Thirty-six healthy young males and females were randomized into groups and directed on attentional focus in a staged putting competition scenario intended to elicit competitive anxiety. External focus groups experienced less internal workload at all arousal levels and preserved heart rate variability measures when audiovisual distraction was introduced.

## 1 Introduction

Motor tasks ranging from everyday activities to competitive sports are highly complex processes that are significantly affected by physiological and psychological system dynamics (Mancevska et al., 2016; Mechsner, 2004; Tanaka and Sekiya, 2010). During activities such as sports competition, where situational incentives such as social comparison and rewards for success drive pressure to perform, psychological factors become more influential (Beilock and Gray, 2007; Martens, 1975). Per the processing efficiency theory (Eysenck and Calvo, 1992), performance changes resulting from this pressure to perform are due to the effects of anxiety on the performer’s limited attentional capacity. Superior performance requires efficient and effective resource allocation across the entire human operating system (Amico and Schaefer, 2022; Delignières et al., 1994; Englert and Bertrams, 2013). Englert and Bertrams (2013) found that temporarily depleting self-control in a group of participants resulted in a negative relationship between anxiety and perceptual motor performance that wasn’t found in the non-manipulated self-control group. Williams, Vickers, and Rodrigues found that performance among table tennis players was diminished while self-reported effort, number of eye fixations, and probe reaction time were all increased in high anxiety conditions (Williams et al., 2002). These changes in fixation and reaction time with increased anxiety exemplify less efficient processing, which the processing efficiency theory points to as a driving factor in performance.

The constrained-action hypothesis states that external focus of attention, or focusing attention on the effects of the intended movement, when conducting a perceptual motor task has been shown to positively affect both learning and overall performance (Wulf et al., 2001b). Furthermore, external focus in skilled performers has been shown to overcome the potentially negative effects of competitive anxiety (Bell and Hardy, 2009). It has been theorized that the benefits of external focus result from encouraging automatic processes of movement to occur, which lessen depletion of attentional resources compared to allowing for optimized learning and performance (Wulf et al., 2001a). While some argue that the constrained-action hypothesis applies solely to skilled performers (Castaneda and Gray, 2007), Munzert et al. (2014) showed that unskilled performers who maintained an external focus displayed more ideal putting kinematics than those maintaining an internal focus.

The lowered processing efficiency observed with increased anxiety can also be observed via measures of cardiac physiology. The autonomic nervous system is responsible for controlling systemic resource allocation in preparation for and recovery from motor performance workloads (Stephenson et al., 2021). This is performed via regulation of the parasympathetic (rest and digest) and sympathetic (fight, flight, or freeze) nervous systems. This interplay causes minute temporal variation in cardiac dynamics, also known as heart rate variability (HRV). For example, assessing the low (0.04 Hz–0.14 Hz) frequency band reflected self-reported mental effort levels, while changes in the high (0.15 Hz–0.4 Hz) frequency band were associated with anxiety-based performance impairment (Mullen et al., 2005). These changes in autonomic tone in response demonstrate the potential to analyze individual measures of HRV to extrapolate an individual’s physiological response to a psychological facet: anxiety.

There have not been adequate efforts to explore the physiological effects of competitive anxiety and secondary distractors on externally focused performers. Pelleck and Passmore (2017) utilized surface electromyography (sEMG) during putting with various attentional foci, finding that the skill level of the practitioner and distance of focus from the key elements of the motor skill in question affect the execution of said motor task. Castaneda and Gray (2007) had similar findings, though with both skilled and novice baseball players in a batting simulation. While this clarifies the understanding and optimization of performance on a motor task, the degree to which these same findings occur as arousal increases needs to be investigated.

The objective of this study was to explore the effects of competitive anxiety and distraction stimuli on externally focused performers, which has the potential to provide key insights into resource allocation and attentional focus while performing under anxiety.

We hypothesized that putting performance would be greater in those participants directed to maintain an external focus as opposed to those given no attentional focus, across pressure conditions. We conjectured that increasing attentional demands via competitive stress would be accompanied by a greater increased internal workload, measured via heart rate, in the non-directed attentional focus groups than the external attentional focus groups. We also speculated that the addition of audiovisual distraction, further increasing anxiety, would result in greater internal workloads than their non-distraction group counterparts.

## 2 Materials and methods

### 2.1 Participants

Participants were 36 (*n* = 22 males and *n* = 14 females) adults recruited from the local area, aged between 18 and 45 years (Mean = 23.5 years, SD = 5.9 years). Recruitment was performed via campus email, public facing fliers placed at local minigolf and golf courses, social media, and word of mouth. Exclusion criteria for this study consisted of being under the age of 18 or above the age of 65, pregnant, suffering a balance deficiency, have a history of hemiparesis or hemiplegia, or a history of Bell’s palsy or other cranial nerve dysfunction. All study procedures were approved by the West Virginia University Institutional Review Board (AAHRPP Accredited). All participants provided their informed consent before participating in any study related activity.

### 2.2 Equipment

All trials were performed on a level, green AstroTurf running track. An 11 cm (4.33 in) diameter practice putting hole was secured to the track, and a marker of tape indicating the putting position was secured to the track six feet (1.83 m) from the participant facing edge of the putting hole. This distance was found during pilot testing to provide a 30% success rate in self-perceived novice putters, and a 75–85% success rate in self-perceived experienced putters. Two putting irons were provided (an 83.82 cm Odyssey White Steel 2 Ball CS Mallet Putter and an 86.36 cm Spalding Pro Series Ps-2 Blade Putter), though participants were informed that they were allowed to bring their own. Participants were allowed to self-select putting iron, including time to practice with either choice. Standard golf balls were used by all participants.

Shot timing was recorded via the Emerald Timestamp app (Emerald Sequoia LLC). This application utilizes Network Time Protocol to sync with networked computer systems automatically. Heart rate was captured via Polar (Polar Electro) H10 heart rate monitor chest strap connected by Bluetooth to a Polar Grit X watch which was in turn synced via Network Time Protocol before each data collection session. This allowed an accurate comparison of putting time with the heart rate to the nearest second, as the sampling rate of the H10 heart rate monitor when paired with the Polar Grit X watch is 1 kHz. Following manufacturer specifications, participants were instructed to wear the H10 chest strap, with the electrode area of the strap lightly moistened, and centered approximately 3.81 cm below the xiphoid process. The activity mode selected on the Polar Grit X watch for the duration of data collection was “Running.”

Neurocognitive tasks, anxiety and workload questionnaires, and trait and experience surveys were all administered via an in-house developed application on a laboratory-provided computer tablet.

### 2.3 Design

Participants were tested individually over the span of an hour. Acclimation putts were included to control for learning effect. While no maximum putt limit was placed on the acclimation phase, participants were asked to perform at least 15 putts. The mean number of putts before participants indicated readiness to proceed was 21.3 putts. Two testing blocks were performed, during which participants completed three trials of ten putts each (1 trial = 10 putts). The first testing block was the low stress condition, and the second testing block was the high stress condition. In total, the participants completed 30 putts in the low stress condition and 30 putts in the high stress condition.

In the low stress condition trials, participants were informed that the results of these putts did not matter, would not be used for their score in the competition, and were only to ensure that the heart rate monitors were working correctly during putting. During this time the study personnel assumed an intentionally light and supportive manner.

Following completion of the low stress condition trials, participants were briefed about the high stress trials, presented in the form of a faux competition. A falsified leaderboard consisting of five commonly encountered first names and final scores ranging between 10 and 26 was presented to the participant. Tally marks of misses and hits from an imaginary “last participant” were erased from the scoring whiteboard in view of the current participant. The instructions indicated that the total number of successful putts for the next three trials would be tallied, and that the participant had 2 min to perform each individual trial. Participants were told their name would be added to the leaderboard and replace one of the prior participants if the score was high enough.

At the completion of each trial, hit and miss tally marks were added to the score board and the participant was verbally judged on their performance. If the performance was less than 40% of putts made, the phrasing was doubtful of the participants’ ability to achieve a leaderboard score. If the performance was at least 40% of putts made, the phrasing was a reminder not to “choke.”

Upon completion of data collection, participants were informed of the deception involving competition. They were informed that all participant names and scores on the leaderboard were falsified to create competitive anxiety, and that their name would not be made available to any other participants. All debriefing followed a script approved by the Institutional Review Board.

## 3 Experimental groups

Participants were randomly distributed between four groups, resulting in n = 9 for each group. These groups consisted of a nondirected focus without distraction group, an external focus without distraction group, a nondirected focus with audiovisual distraction, and an external focus with audiovisual distraction group. The non-directed focus groups were given no instruction on attentional focus, while the external focus groups were instructed to focus their attention on the desired outcome of the movement: the golf ball going into the hole. These instructions were repeated before the onset of each trial in both conditions.

During the high stress condition trials, the non-distraction groups were not subjected to audiovisual distraction, while the non-directed focus/distraction and the external focus/distraction groups were exposed to approximately 75 dBA crowd noise played from a Bluetooth speaker positioned facing the participant and visual distraction in the form of study personnel waving a white beach towel through the air directly behind the target hole. A breakdown of group demographics can be found in Table 1.

**Table 1:** Group demographic information.

Group	Attentional focus	Distraction status	Sex	Mean age	Experience (Years)
1	Nondirected	Nondistract	3 F, 6 M	23	4.2
2	External	Nondistract	3 F, 6 M	27	10.4
3	Nondirected	Distract	3 F, 6 M	23.3	9.1
4	External	Distract	6 F, 3 M	20.7	8.1

## 4 Measures

### 4.1 Performance

Performance was measured via the binary outcome of each putt, recorded by study personnel. Participants were not informed that performance was being assessed in the low stress condition but were aware in the high stress condition.

### 4.2 Competitive state anxiety

Before each trial, the participants engaged in the Competitive Stress Anxiety Inventory-2 Revised (CSAI-2R), a sport specific 17-question inventory which has been shown to be a reliable and valid measure of three facets of competitive stress: cognitive anxiety, somatic anxiety, and self-confidence (Cox et al., 2003). Alpha reliability coefficients range from 0.70 to 0.90 (Martens et al., 1990). Participants rated their current anxiety on a Likert scale ranging from one (*not at all*) to four (*very much so*).

### 4.3 Cardiac variables

After completion of the informed consent process, participants were supervised in the proper donning of the Polar H10 heart rate monitor and instructed to lay in a supine position with eyes closed and without talking for a 10-min heart rate baseline with HRV measured the last 5 min of resting. Heart rate and interbeat interval (IBI), or the time between subsequent heartbeats, were measured throughout the entire data collection process. Pre-trial HRV epochs consisted of 60 s beginning after the completion of the CSAI-2R and ending with the start of each trial.

Cleaning and analysis of IBI data from the Polar H10 heart rate monitor was performed via Kubios, a validated and widely utilized research tool for the calculation of heart rate variability variables (Kubios HRV program, Biosignal Analysis and Medical Imaging Group, University of Kuopio, Finland). For this effort, focus was placed on root mean square of successive differences (RMSSD), which is generally utilized to estimate parasympathetic activity (Dong, 2016; Stephenson et al., 2021). Specifically, HRVRMSSD was calculated as the change from resting baseline to pre-trial RMSSD.

### 4.4 Perceived effort

At completion of each trial, participants completed the NASA Task Load Index (NASA-TLX), which weighs six subscales based on participant evaluation of contribution to workload and creates an overall perceived workload score (Hart and Staveland, 1988). The NASA-TLX is a widely used measure with alpha reliability coefficient greater than 0.80 (Xiao et al., 2005). Participants rated each subscale on a Likert scale ranging from one (*low*) to twenty (*high*).

## 5 Results

### 5.1 Statistical analysis

All analyses were carried out with R version 4.3.2 (the R foundation for Statistical Computing, Vienna, Austria). For analysis of the low stress condition, where no audiovisual distraction was present, the four groups were combined into two groups of 18 participants: nondirected focus and external focus. Welch’s t-test was then applied for anxiety, performance, cardiac variables, and perceived workload in the low stress condition. The introduction of audiovisual distraction in the high stress condition led to the use of a two factor ANOVA based on presence of audiovisual distraction and attentional focus.

### 5.2 Manipulation check

To evaluate the efficacy of the arousal manipulation, both heart rate and competitive anxiety scores for all groups combined were compared between the low stress and high stress conditions via Welch’s t-test. Mean heart rate in the low stress condition was 44.95 bpm and rose in the high stress condition to 50.74 bpm (*p* = 0.0005). For cognitive anxiety, mean scores rose from 7.82 to 9.02 in the low stress and high stress conditions, respectively (*p* = 0.0046). Taken together, this indicates that the competitive pressure manipulation was effective.

### 5.3 Arousal condition

**Figure 1:**
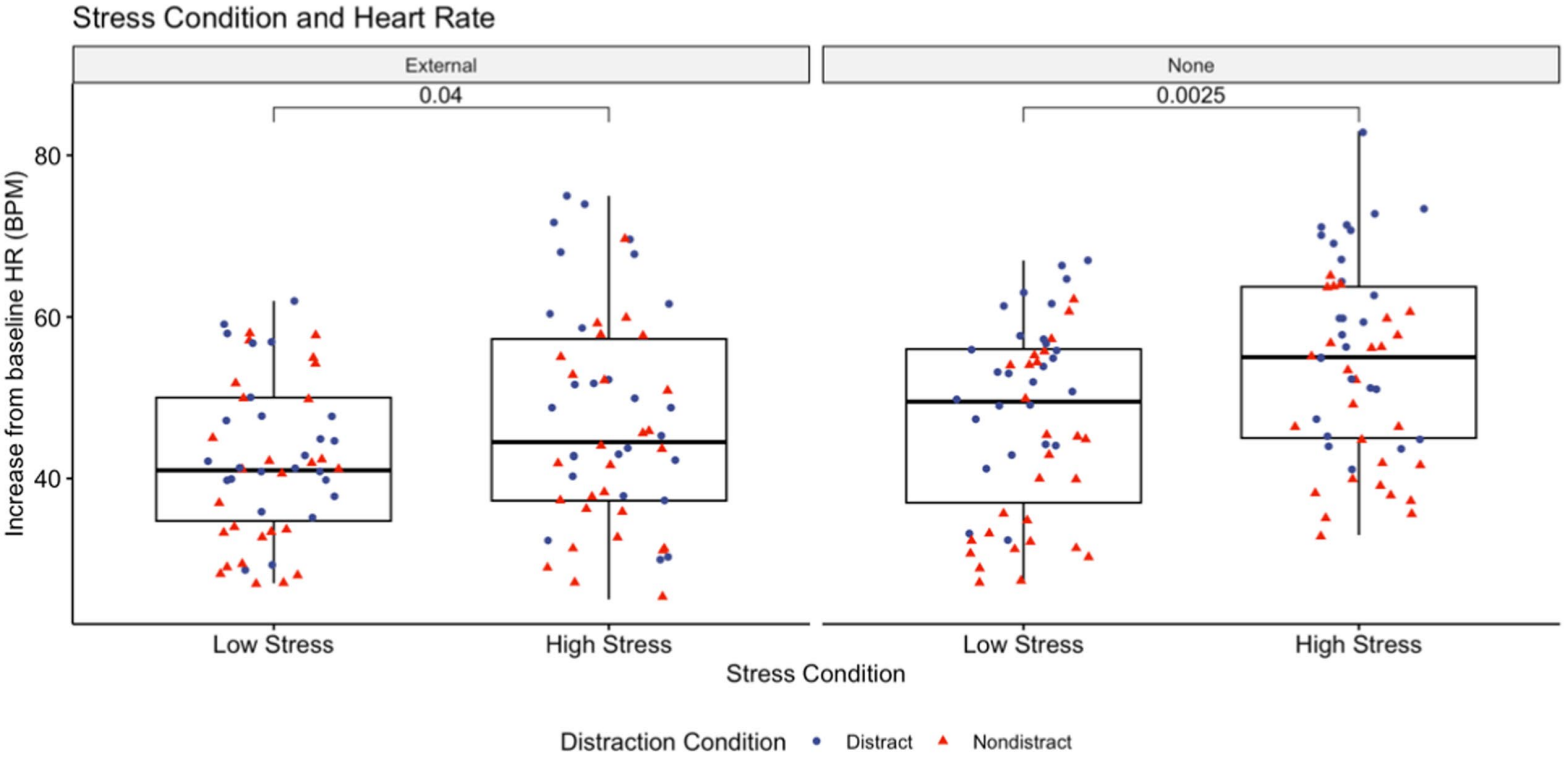
Demonstrates the rise in internal workload, measured via heart rate, with the addition of competitive anxiety among both attentional focus conditions. Cohen’s D for this comparison in the external focus and non-directed focus groups were −0.404 (small) and −0.597 (moderate), respectively.

A significant main effect for stress condition confirmed that for all groups combined cognitive and somatic anxiety levels, as well as change in heart rate from resting baseline, increased between the low stress and high stress conditions (Figure 1). Mean (±SD) values for change in heart rate and heart rate variability from baseline, cognitive and somatic anxiety, self-confidence, and perceived workload for both low stress and high stress conditions are shown in Table 2. Summary statistics for anxiety, performance, cardiac variables, and perceived workload in the high stress condition can be found in Table 3. Putting performance was significantly affected by the addition of competitive pressure, increasing from a mean trial score of 4.57 in the low stress group and 5.19 in the high stress group (*p* = 0.023). This may be misleading, however, as only one of the four groups: the no attentional focus, no distraction group, was significantly different when comparing intragroup low-pressure scores (mean = 4.81) to high-pressure scores (mean = 6.44, *p* = 0.0055). The three other groups showed no difference in score between arousal conditions.

**Table 2:** Means (±SD) for anxiety, performance, cardiac, and workload variables.

Variable	Distraction status	Low stress condition		High stress condition	
		Attentional focus		Attentional focus	
		None	External	None	External
Score	Distract	4.59 (2.02)	4.15 (1.66)	4.64 (1.75)	4.78 (1.45)
	Nondistract	4.82 (2.15)	4.74 (2.28)	6.58 (1.70)	4.89 (2.24)
Cognitive anxiety	Distract	8.0 (2.50)	8.04 (3.09)	8.4 (2.78)	10.2 (4.06)
	Nondistract	8.93 (2.53)	6.33 (1.82)	10.1 (2.64)	7.41 (3.08)
Somatic anxiety	Distract	8.33 (1.84)	8.0 (1.30)	10.6 (3.45)	10.4 (2.68)
	Nondistract	12.7 (4.09)	8.93 (2.22)	14.1 (3.55)	9.85 (3.36)
Self-confidence	Distract	15.1 (3.24)	13.4 (2.57)	15.6 (5.06)	12.3 (2.70)
	Nondistract	13.4 (3.56)	12.8 (3.25)	14.3 (3.66)	13.3 (4.34)
Perceived workload	Distract	16.4 (4.58)	12.7 (4.83)	19.3 (4.93)	14.7 (6.68)
	Nondistract	17.3 (6.86)	14.1 (4.86)	24.2 (8.97)	16.5 (4.02)
Heart rate	Distract	52.5 (9.11)	44.5 (8.84)	59.2 (11.2)	51.1 (13.7)
	Nondistract	42.0 (11.4)	40.7 (10.4)	49.3 (10.4)	43.4 (11.8)

**Table 3:** ANOVA summaries for all variables in high stress condition.

Variable	High stress condition		
	df	*F*	*η*^2^
Score			
Focus	1, 104	5.040*	0.042
Stimuli	1, 104	7.962**	0.066
Focus x Stimuli	1, 104	6.660*	0.055
Cognitive anxiety			
Focus	1, 104	0.052	0.000
Stimuli	1, 104	0.965	0.008
Focus x Stimuli	1, 104	15.716***	0.133
Somatic anxiety			
Focus	1, 104	12.308***	0.096
Stimuli	1, 104	5.090*	0.04
Focus x Stimuli	1, 104	9.968**	0.078
Self-confidence			
Focus	1, 104	7.348**	0.066
Stimuli	1, 104	0.014	0.000
Focus x Stimuli	1, 104	2.352	0.021
Perceived workload			
Focus	1, 104	24.320***	0.182
Stimuli	1, 104	6.964**	0.052
Focus x Stimuli	1, 104	1.584	0.012
Heart rate			
Focus	1, 104	9.459**	0.074
Stimuli	1, 104	14.874***	0.116
Focus x Stimuli	1, 104	0.238	0.002
HRVRMSSD			
Focus	1, 104	8.037**	0.071
Stimuli	1, 104	1.696	0.015
Focus x Stimuli	1, 104	0.012	0.000

*Significant at *p* < 0.05; **significant at *p* < 0.001; ***significant at *p* < 0.0001.

### 5.4 Attentional focus

**Figure 2:**
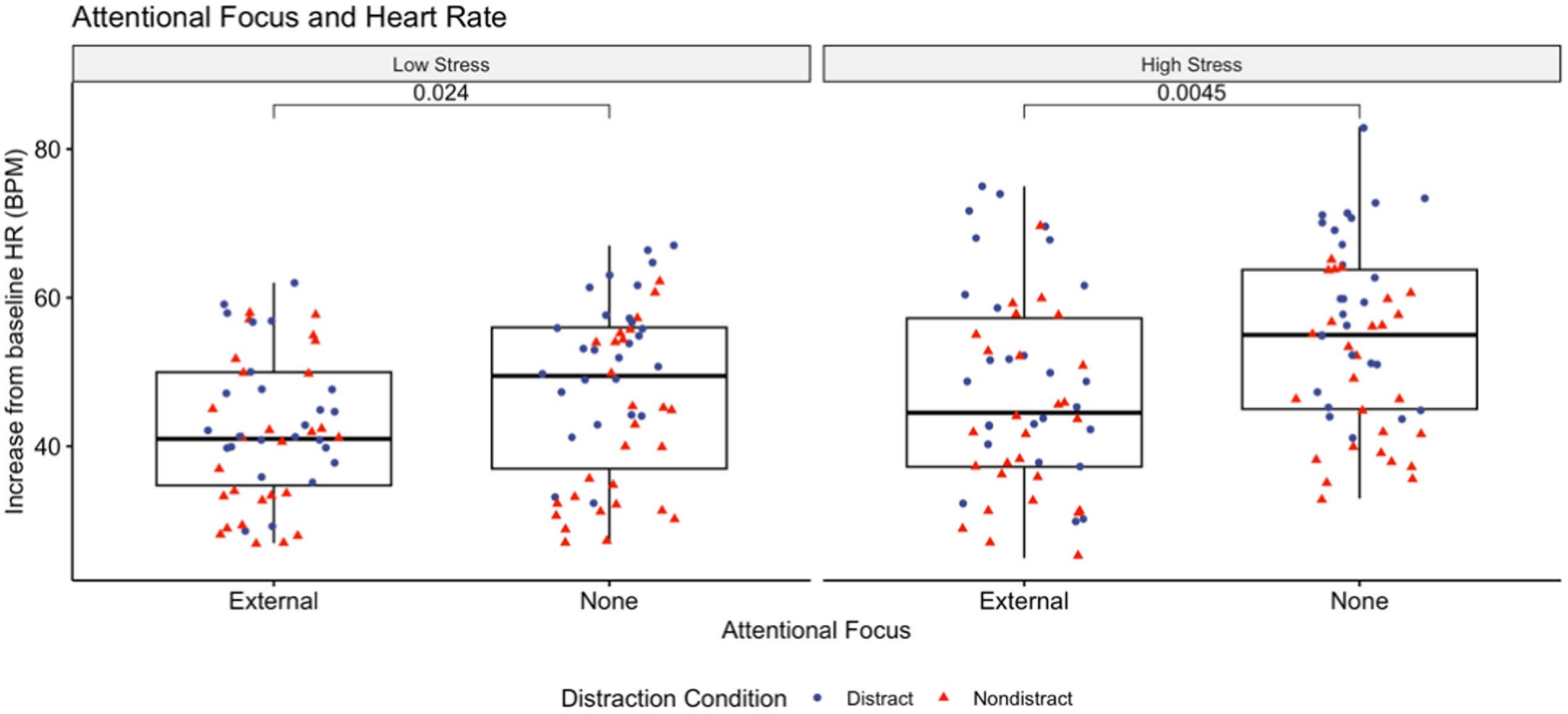
Demonstrates the difference in internal workload, measured via heart rate, for both attentional focus conditions at both competitive stress levels. At a higher level of competitive stress, the participants who maintained an external focal point had smaller increases from baseline. Cohen’s D for this comparison in the low stress and high stress conditions were −0.444 (small) and −0.558 (moderate), respectively.

**Figure 3:**
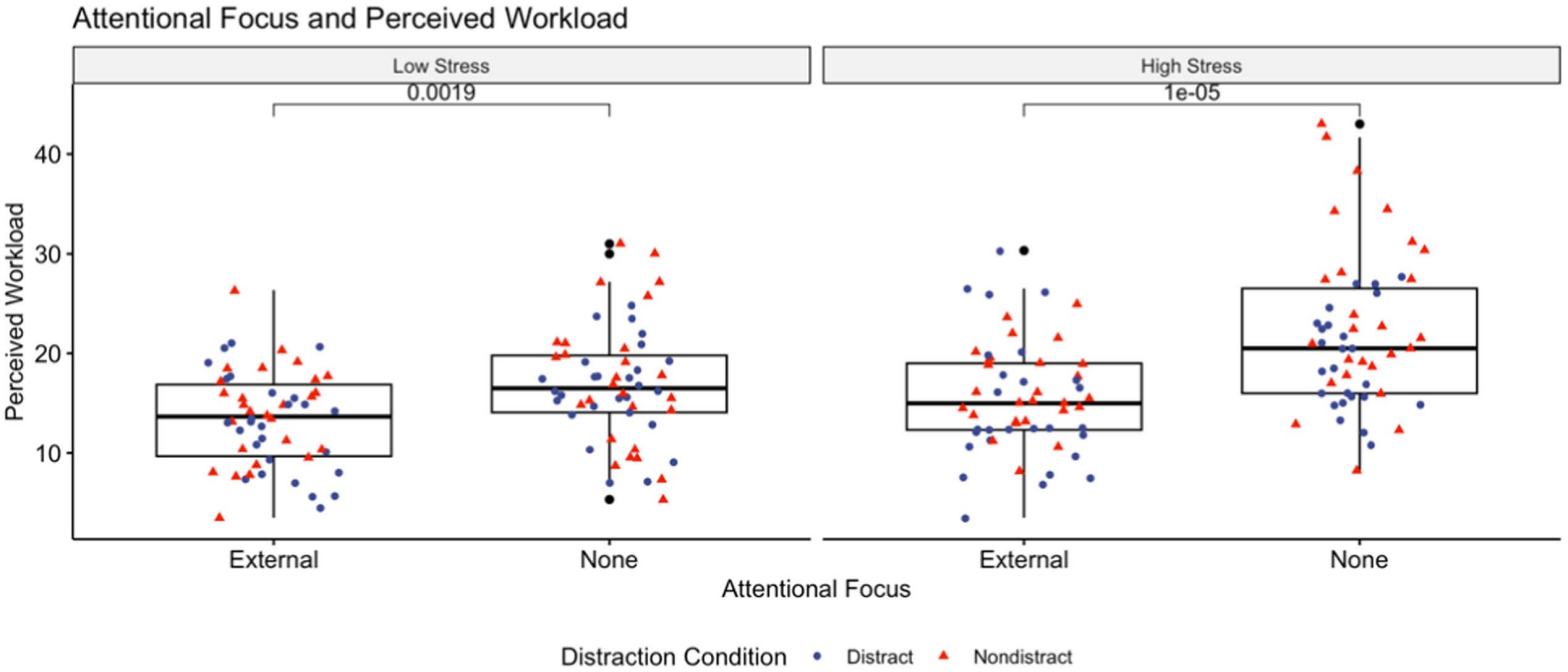
Demonstrates the difference in perceived workload, measured via NASA-TLX, for both attentional focus conditions at both competitive stress levels. At a higher level of competitive stress, the participants who maintained an external focal point had operated with lower perceived effort. Cohen’s D for this comparison in the low stress and high stress conditions were −0.642 (moderate) and −0.929 (large), respectively.

Mean (±SD) values for change in heart rate and heart rate variability from baseline, cognitive and somatic anxiety, self-confidence, and perceived workload for both non-directed and external attentional focus are shown in Table 4. As is visible in Figure 2, increase in heart rate from baseline was significantly lower in the external attentional focus groups in both the low stress and competition arousal conditions. While absolute change from baseline in RMSSD was greater, cognitive and somatic anxiety were significantly lower in external attentional focus groups than non-focus directed groups. Perceived workload was also significantly lower in the external attentional focus groups for both arousal conditions, which can be seen in Figure 3.

**Table 4:** Means(±SD) for all variables for non-directed and external attentional focus.

Variable	Attentional focus	Stress condition	
		Low stress	High stress
Score	External	4.44 (2.00)	4.83 (1.87)
	None	4.70 (2.07)	5.63 (1.97)
Cognitive anxiety	External	7.18 (2.66)	8.80 (3.83)
	None	8.46 (2.53)	9.26 (2.81)
Somatic anxiety	External	8.46 (1.86)	10.1 (3.02)
	None	10.5 (3.82)	12.4 (3.90)
Self-confidence	External	13.1 (2.92)	12.8 (3.62)
	None	14.3 (3.48)	14.9 (4.41)
Perceived workload	External	12.8 (2.86)	13.0 (3.30)
	None	14.7 (4.06)	15.0 (4.02)
Heart rate	External	42.5 (9.78)	47.2 (13.2)
	None	47.2 (11.5)	54.2 (11.8)
RMSSD	External	31.5 (33.6)	30.9 (33.5)
	None	17.2 (16.9)	16.3 (18.0)

### 5.5 Audiovisual distraction

**Figure 4:**
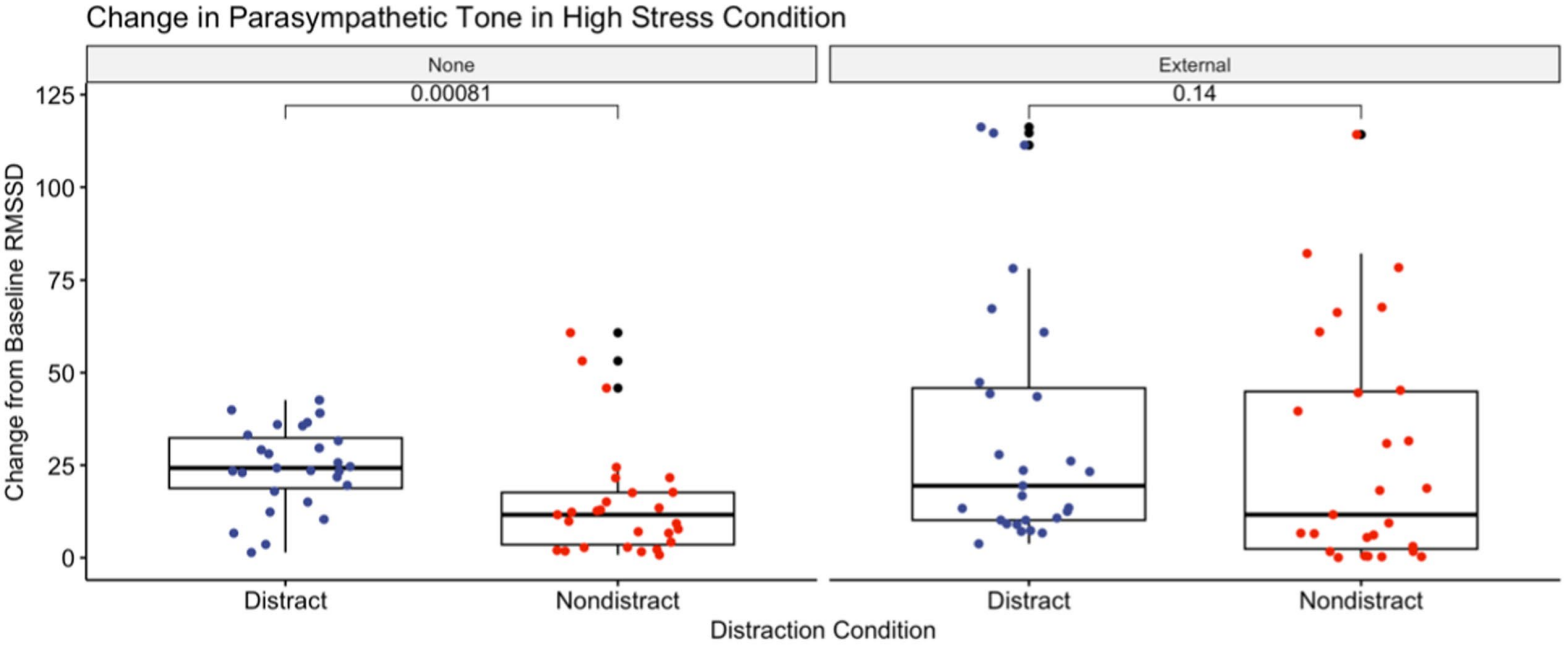
Demonstrates the difference in parasympathetic tone, measured via the RMSSD aspect of heart rate variability, for both attentional focus conditions and distraction conditions in the high competitive anxiety condition. Greater change from baseline RMSSD indicates a greater withdrawal of vagal tone. There was no significant difference in change from baseline RMSSD between distraction conditions for participants who were coached to maintain an external attentional focus point. This was false for those who were not coached to maintain an external attentional focus, with the distraction group showing a greater loss of parasympathetic tone from baseline. Cohen’s D for this comparison was −0.546 (moderate).

As the distraction manipulation was not present in the low stress condition, only data from the high stress condition are being utilized for these comparisons. Mean (±SD) values for change in heart rate and heart rate variability from baseline, cognitive and somatic anxiety, self-confidence, and perceived workload for both non-distracted and audiovisual distraction conditions are shown in Table 5. Putting performance was significantly better in the non-distracted condition than in those experiencing audiovisual distraction, indicating a successful manipulation. Also of note, change in baseline from RMSSD was significantly greater for participants undergoing an audiovisual distraction than for those with no distraction, but only in those participants who were not directed on attentional focus. Those who were instructed to maintain an external attentional focus showed no difference between presence or lack of audiovisual distraction (Figure 4).

**Table 5:** Means (±SD) for all variables for non-distraction and audiovisual distraction.

Variable	Distraction status	Stress condition	
		Low stress	High stress
Score	Distract	4.37 (1.85)	4.72 (1.57)
	Nondistract	4.78 (2.19)	5.67 (2.16)
Cognitive anxiety	Distract	8.14 (2.77)	8.87 (3.30)
	Nondistract	8.43 (2.80)	8.79 (2.94)
Somatic anxiety	Distract	8.92 (2.58)	9.73 (2.87)
	Nondistract	11.9 (3.99)	12.2 (4.04)
Self-confidence	Distract	14.7 (3.81)	14.3 (4.0)
	Nondistract	13.5 (3.5)	13.7 (3.84)
Perceived workload	Distract	13.6 (3.05)	13.7 (3.80)
	Nondistract	13.2 (3.71)	13.4 (3.78)
Heart rate	Distract	52.3 (11.4)	54.3 (11.9)
	Nondistract	44.0 (11.3)	44.9 (11.5)
RMSSD	Distract	25.2 (25.5)	25.2 (25.1)
	Nondistract	18.1 (24.0)	17.7 (24.0)

## 6 Discussion

The first hypothesis, that putting performance would be greater with the increase in arousal in those groups maintaining an external focus, was not supported by the findings of this study. As noted in the Results section, only one of the four groups had significant differences in score between the low and high stress conditions: the non-directed attentional focus/non-distraction group. We believe that while the competition stages elicited higher internal workloads in both focus groups, represented by an increase in average heart rates, the demands of the task were not high enough to deplete available cognitive resources and allow for observation of performance changes. Additionally, preservation of performance may have been achieved via increases in effort, supported by increased internal and perceived workloads with increased arousal (Eysenck et al., 2007). It is also possible that our mostly novice participants would have benefited from a more proximal attentional focus than the ball going into the hole, for example focusing on causing the ball to pass through an imaginary mark one foot away, on the path from the starting location to the golf hole (Wulf and Su, 2007).

The second hypothesis, that internal workload demands would be greater in the non-focus directed group than the external focus group, was upheld by the data. Change in heart rate from baseline was significantly higher in the non-directed focus group than in the external focus group in both arousal conditions of the study. In addition, score was not significantly different between focus groups in either of the arousal conditions. This indicates a compensatory increase in internal workload to achieve the same performance, regardless of arousal condition, due to attentional focus. This, combined with higher perceived workload in non-directed focus groups lends support to Wulf’s constrained action hypothesis (Wulf et al., 2001a).

As predicted, the inclusion of audiovisual distraction resulted in a significant difference in change in heart rate from baseline in the high stress condition. This speaks to the spread allocation of resources required in the presence of task distraction, regardless of attentional focus. Of note was the lack of change, or “rescue,” of parasympathetic tone seen with the addition of audiovisual distraction in the external focus group. While both the non-directed and external attentional focus groups saw decreases in vagal tone from baseline, only the non-directed attention focus group had significant difference in vagal tones, with those experiencing audiovisual distraction more affected than those experiencing no distraction. The presence of said preservation may indicate that an external attentional focus has a mitigating effect on arousal while performing a motor task (Figure 4).

A major limitation of this study was the equal task design. Further investigation utilizing skill-based task demands, such as larger or smaller holes depending upon participant performance during the orientation stage, could possibly elicit the performance declines that were originally hypothesized. To make the task accessible for the potential participant base, pilot testing resulted in a less difficult putting task than originally planned (e.g., 10-foot distance). Additionally, the use of a short questionnaire to identify the participants’ attentional focus, whether internal or external, after each stage of putting could have resulted in more accurate comparison groups. This could alleviate concerns that some members of the non-focus directed group may have utilized an external focus, though prior research does show that non-focus directed groups consistently produce identical results to internal focus directed groups (Wulf and Su, 2007). The homogeneous nature of age in our study population could possibly be seen as a limitation, but because this work was not meant to investigate the effects of age on competitive anxiety and motor task performance, the authors feel this is not a hinderance to the goals of the study. An additional weakness is the low number of participants, though the number of participants per group is similar to prior studies in the field (Hardy et al., 1996; Mullen et al., 2012).

In conclusion, participants in both attentional focus groups saw no degradation in performance, regardless of arousal condition. In the external attentional focus groups, preservation of performance was achieved while utilizing less physiological resources than in the non-directed attentional focus group, even in the presence of audiovisual distraction. External attentional focus-based strategies may therefore be of use at all skill levels, and for multiple purposes: learning proper motor patterns, coping with competitive anxiety, performing in the presence of distraction, and fatigue management.

## Data Availability

The raw data supporting the conclusions of this article will be made available by the authors, without undue reservation.
